# Role of the Rostral Ventrolateral Medulla in the Arterial Hypertension in Chronic Renal Failure

**DOI:** 10.4061/2010/219358

**Published:** 2011-01-04

**Authors:** Adriana P. Castilho Dugaich, Elizabeth B. Oliveira-Sales, Nayda P. Abreu, Mirian A. Boim, Cássia T. Bergamaschi, Ruy R. Campos

**Affiliations:** ^1^Cardiovascular Division, Department of Physiology, Federal University of São Paulo, São Paulo, SP, Brazil; ^2^Nephrology Division, Department of Medicine, Federal University of São Paulo (UNIFESP), São Paulo, SP, Brazil; ^3^Cardiovascular Division, Department of Physiology, Federal University of São Paulo, Sao Paulo School of Medicine, Rua Botucatu, 862, 04023-060 Sao Paulo, SP, Brazil

## Abstract

Sympathetic activation in chronic renal failure (CRF) is a major mechanism leading to the progression of renal disease and hypertension. In the present study, we tested the hypothesis that in CRF increased reactive oxygen species (ROS) production in the RVLM mediated by enhanced circulating Angiotensin II (Ang II) is an important mechanism leading to hypertension in CRF. In CRF rats we found an increase in the abundance of p47^phox^ and gp91^phox^ mRNA within the RVLM associated with a reduction of Ang II type 1 receptors (AT_1_) mRNA in the brainstem compared to controls (C). Tempol but not candesartan into the RVLM decreased MAP in CRF but not in C rats. GABA into the RVLM decreased MAP in CRF (63 ± 8 mmHg) more intensely than in C (33 ± 3 mmHg). The results suggest that increased oxidative stress within the RVLM has an important participation to maintain hypertension in CRF rats apparently independently of AT_1_ Ang II receptors.

## 1. Introduction

The chronic renal failure (CRF) is characterized by high circulating Angiotensin II (Ang II) that causes direct vasoconstriction, increases aldosterone secretion, enhances sympathetic nerve activity, and increases reactive oxygen species production (ROS), acting at peripheral and central sites [1]. 

There is increasing evidence to support the hypothesis that ROS play a major role in the pathophysiology of CRF [2]. In fact, Ang II action in the brain increases the activity of NAD(P)H oxidase, a major source of superoxide anion (O_2_) production [2]. NAD(P)H oxidase is composed of two membrane-bound subunits (gp91^phox^ and p22^phox^), several cytoplasmic units (p40^phox^, p47^phox^, and p67^phox^), and the small G protein Racla [3]. After activation of Ang II type 1 (AT_1_) receptors, the cytoplasmic subunits bind to the membrane subunits and activate the enzyme, resulting in the intracellular production of O_2_^−^ [4]. Therefore, the first aim of the present study was to quantify the NADPH p47^phox^ and gp91^phox^ subunits expression within the RVLM. Considering that in CRF there is an increase in the brain Ang II, the expression of AT_1_ subtype receptor was also quantified in the RVLM.

To functionally test the role of oxidative stress and AT_1_ receptors on hypertension in CRF rats we injected into the RVLM the superoxide mimetic-tempol and the AT_1_ antagonist-candesartan, respectively. Finally, the importance of RVLM in the maintenance of hypertension in CRF was evaluated by injection of GABA into the region.

## 2. Materials and Methods

### 2.1. General Procedures

All experimental procedures were conducted according to the National Institutes of Health guidelines for use and care of animals, and the study protocol was approved by the Ethics in Research Committee of the Federal University of Sao Paulo School of Medicine (process no. 0661/04).

Male Wistar rats (*n* = 55, 250 to 300 g) were obtained from the animal care facility of our institution. The animals were housed in group cages, given access to rat chow and water *ad libitum*, and maintained in a temperature-controlled environment (23°C) on a 12-h light/dark cycle.

The drugs urethane (Ethyl carbamate), glutamate, GABA, tempol, candesartan, hexamethonium, and potassium chloride from Sigma (USA) were all dissolved in saline. And Xylazine (Sedafarm-Farmabase Saúde Animal Lta-Brazil), Ketamine (Agener União-Brazil) were used.

### 2.2. Experimental Protocols

#### 2.2.1. Hypertensive Animals

The animals were anesthetized with Ketamine and Xylazine (40 and 20 mg/Kg, i.p., resp.), the right kidney was removed, and the posterior and the inferior arteries of the left kidney were tied, consequently leading to the irrigation of the superior portion of the kidney (5/6 nephrectomy). The control group had sham operation. The animals were taken to their home cage after the surgery for recovery, and only after four weeks of the renal surgery they were instrumented so that the experiments could be carried out thereafter.

#### 2.2.2. RNA Isolation, Reverse Transcription, and Quantitative Real-Time PCR

Rats were euthanized by decapitation, brainstem, and cerebellum immediately removed and stored at −80°C. Total RNA was purified from tissue by the phenol and guanidine isothiocyanate-cesium chloride method using an appropriate kit (Trizol, Life Tecnologies, USA). Two micrograms of total RNA were treated with DNase (RQ1 Rnase-Free Dnase, Promega) to prevent genomic DNA contamination. The RNA pellet was resuspended in RNase-free water. Reverse transcribed into cDNA by the addition of a mix containing 0.5 mg/mL oligo d(T), 10 mM DTT, 0.5 mM dNTPs (Pharmacia Biotech), and 200 U of reverse transcriptase enzyme (SuperScript RT, Gibco-BRL). Real-time amplification was obtained using a GeneAmp 5700 Sequence Detection System SDS (ABI Prism 7700, Applied Biosystems, CA, USA). Real-time PCR product accumulation was monitored using the intercalating dye, SYBR Green I (Molecular Probes Inc., USA), which exhibits a higher fluorescence upon binding of double-stranded DNA. Relative gene expression was calculated using conditions at the early stages of PCR, when amplification was logarithmic and, thus, could be correlated to the initial copy number of gene transcripts. The reactions were cycled 40 times under the conditions previously determined by conventional PCR. Fluorescence for each cycle was quantitatively analyzed by ABI Prism 7700 SDS (Applied Biosystems, CA, USA). At the end of the PCR, the temperature was increased from 60 to 95°C at a rate of 2°C/min, and fluorescence was measured every 15 s to construct the melting curve. A nontemplate control (NTC) was run with each assay. The relative amount of each mRNA was estimated using a standard curve constructed from serial dilutions of cDNA including 1/1, 1/10, and 1/100. PCR was performed with primers selective for AT_1_ receptor_,_ p47^phox^, and gp91^phox^ (Table 1). Results of 5 experiments per group are reported as relative expression normalized with the *β*-actin housekeeping gene, used as an endogenous control and expressed in arbitrary units.

#### 2.2.3. Real-Time Data Analysis

The cycle threshold (Ct) values were subtracted from the Ct value for each gene (to give ∆Ct values). These values were used to carry out statistical comparisons. For graphical representation, the fold variation was then determined using the 2^−(ΔΔCt)^ method according to published protocols [5] and manufacturer's recommendations. Fold variation was calculated by determining the difference in ΔCt values between a chosen reference and test sample (∆∆Ct value) and applying the 2^−(ΔΔCt)^ formula.

#### 2.2.4. Surgical Instrumentation for Acquisition of the Cardiovascular Parameters

One day before the experiments, the rats were anesthetized with ketamine and xylazine (40 and 20 mg/Kg, i.p., resp.) and instrumented with femoral venous and arterial catheters, which were constructed from PE-50 and PE-10 tubing filled with heparinized saline, for drug injection and arterial pressure recording, respectively. Catheters were externalized throughout the nape of the animal's neck. After surgery, the rats were returned to their home cage for recovery. All experiments were performed twenty four hours after the surgery. On the day of the experiment, arterial blood pressure (BP), mean arterial pressure (MAP), and heart rate (HR) were recorded in awake, freely moving rats, and animals were then anesthetized very slowly with urethane (1.2–1.4 g/Kg, IV). This procedure was performed to avoid changes in arterial blood pressure in relation to conscious condition during the anesthesia administration. All animals were artificially ventilated with oxygen-enriched air with a respiratory pump. Rectal temperature was maintained at 37 ± 0.5°C by a servo-controlled electric blanket.

The rat was then mounted prone in a stereotaxic frame with the head mildly ventroflexed (bite bar at −11 mm). The occipital bone was opened over the dorsal medulla and caudal cerebellum, and the dura mater was retracted. The anesthetic level was tested during the experiments (withdrawal reflexes to noxious pinching), and extra urethane (0.1–0.5 g/Kg, IV) was given when it was needed.

#### 2.2.5. Drug Microinjections

Glass micropipettes of tip diameter of approximately 20 *μ*m were used to pressure injected L-glutamate (monosodium salt, 10 nmol/100 nL), GABA 50 nmol/100 nL (Sigma), tempol 2.0 nmol/100 nL (Fluka Chemica), candesartan 0.25/0.5/1 nmol/100 nL (Astra Hässle AB-Sweden) into the RVLM. All the solutions were prepared in normal saline (pH 7.0–7.4). Glutamate and other drugs were injected in 5 to 10 seconds sequentially in one side and in the contralateral region; intervals between injection sides were around of 10 seconds. The pipettes were held in a vertical position, and the RVLM was located 3 mm rostral to the *calamus scriptorius*, 1.7-1.8 mm lateral to midline, and 3 mm deep. Injections sites were marked at the end of the experiments by subsequently injecting 50 to 100 nL of Evans blue. In three additional experiments, the vehicle for injected drugs (saline) was injected into the RVLM as a control injection.

#### 2.2.6. Histology

**Figure 1:**
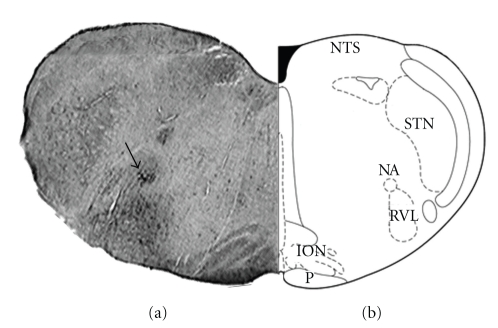
Typical microinjection site in the RVLM evaluated by 100 nL of Evans blue diffusion (a) and schematic representation (b). CST indicates corticospinal tract; ION: inferior olivary nucleus; NA: nucleus ambiguous; NTS: nucleus of the tractus solitari and STN: spinal trigeminal nucleus and P, Pyramid. The arrow indicates nucleus ambiguous.

At the end of the experiments, animals were killed by an overdose of urethane. The brain stem was then removed and fixed by immersion for at least 24 h in 10% paraformaldehyde solution, after which coronal sections (40 *μ*m) were cut on a freezing microtome, and the sections were mounted on glass slices and stained with neutral red. Microinjection sites were identified by deposition of Evans blue.  Figure 1 shows the distribution of the centers of sites at which glutamate (10 nmol/100 nL) was injected into the RVLM. A positive location for the RVLM was considered when the dye was deposited ventral to the nucleus ambiguous and lateral to the inferior olive nucleus.

#### 2.2.7. Statistical Analysis

All values are expressed as mean ± SD. The changes in MAP and HR responses during RVLM microinjections were analyzed in intervals of five seconds after microinjection in relation to basal condition by paired and unpaired *t*-test. The differences on MAP and HR between C and CRF groups were assessed by one-way analysis of variance (ANOVA) followed by the Student-Newman-Keuls method. Differences were considered significant at *P* < .05.

## 3. Results and Discussion

### 3.1. Renal Hypertension

After 4 weeks of renal surgery, there was a significant increase in MAP in renal hypertensive rats compared to control group (CRF, 166 ± 7 mmHg, *n* = 25; C, 111 ± 3 mmHg, *n* = 30). There was no significant change in heart rate (HR) (CRF, 406 ± 13 bpm and C, 403 ± 25 bpm). 

### 3.2. mRNA Brainstem Expression of AT_1_ Receptor and Oxidative Stress Markers in CRF and Control Rats

**Figure 2:**
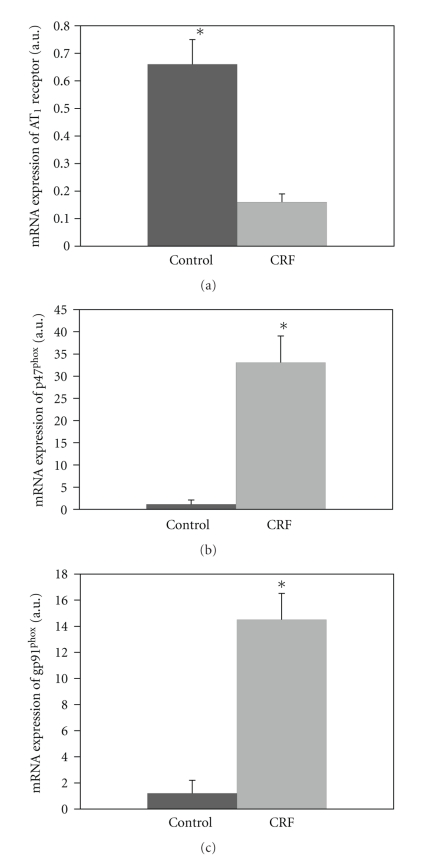
Relative amount of mRNA of AT_1_ in the brainstem (a) and NAD(P)H subunits (p47^phox^ and gp91^phox^) ((b) and (c)) compared to mRNA of *β*-actin of C and CRF groups. *P < .05 difference in relation to the C.

The AT_1_ mRNA expression in brainstem tissue was significantly decreased in CRF rats compared to C group (AT_1_-CRF, 0.16 ± 0.03 and C, 0.66 ± 0.09 AU), as shown in Figure 2(a). According to the real-time PCR, the level of p47^phox^ and gp91^phox^ gene expression in the RVLM in the CRF group was significantly higher than in the C group ((p47^phox^-CRF, 33.07 ± 5.47 and C, 1.13 ± 0.09 AU; *P* < .004) and (gp91^phox^, 14.51 ± 1.49 and C, 1.21 ± 0.28 AU; *P* < .001)), as shown in Figures 2(b) and 2(c). 

### 3.3. Role of Oxidative Stress within the RVLM in Renal Hypertension Evaluated by Tempol Administration

**Figure 3:**
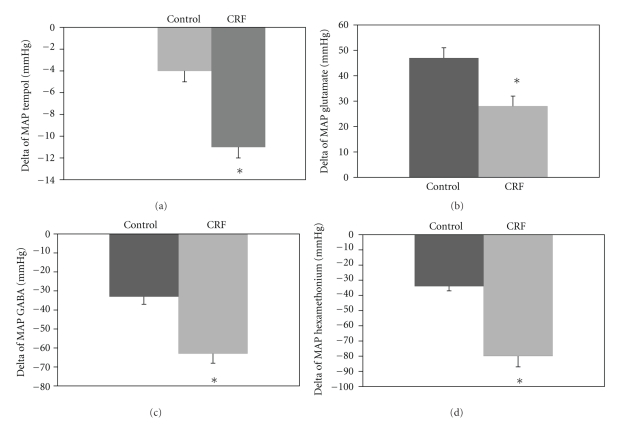
Values of delta of variation of mean arterial pressure (MAP) during bilateral microinjection of tempol (2.0 nmol/100 nL) (a), glutamate (10 nmol/100 nL) (b), GABA (50 nmol/100 nL) (c), hexamethonium (30 mg/kg, IV) (d) into the RVL in the C and CRF groups. *P < .05 difference in relation to the C.

Tempol microinjected into the RVLM decreased MAP in CRF rats (from 164 ± 8 to 153 ± 8 mmHg, *n* = 6) as shown Figure 3(a). In control rats, Tempol into the RVLM did not change MAP.

### 3.4. Role of the AT_1_ Angiotensin II Subtype Receptors into the RVLM in Renal Hypertension

The AT_1_ Ang II antagonist candesartan was bilaterally microinjected into the RVLM (0.25/0.5/1.0 nmol/100 nL) in CRF and C animals. In the three different doses, candesartan significantly increased MAP in both groups, as shown Table 2. The pressor response to candesartan was fast and short-lasting.

### 3.5. Effects of Glutamate and GABA Microinjection into the RVLM in CRF Rats

Bilateral microinjection of glutamate into the RVLM in CRF increased BP (from 166 ± 7, to 194 ± 8 mmHg) (*P* < .05) and decreased HR (from 406 ± 13, to 389 ± 11 bpm, *P* < .05). The same dose of glutamate into the RVLM in C rats caused a significant and more intensive increase in MAP (from 111 ± 3 to 158 ± 5 mmHg) (Figure 3(b)). GABA microinjection into the RVLM in CRF decreased MAP (from 150 ± 5 to 86 ± 8 mmHg, *P* < .05), accompanied by a significant decrease in HR (from 410 ± 13 to 375 ± 17 bpm) (Figure 3(c)). The depressor response to GABA started during microinjection and returned to basal level approximately 14 ± 1.5 minutes after the injection.

In control normotensive rats, the same dose of GABA decreased MAP (from 111 ± 4 to 77 ± 4). The depressor response to GABA started during microinjection and returned to basal level around 6 ± 2.4 minutes after the injection.

The fall in MAP in response to GABA into the RVLM was significantly larger in CRF compared to C animals (MAP 63 ± 8 and 33 ± 3 mmHg, resp. *P* < .05). Hexamethonium administration caused the same pattern of blood pressure reduction as observed with GABA, as shown Figure 3(d).

In the present study, we found a reduction in the mRNA AT_1_ expression in the brainstem tissue in CRF rats accompanied by a significant increase in the level of p47^phox^ and gp91^phox^ gene expression in the RVLM compared to C group. Additionally, tempol but not candesartan into the RVLM caused a decrease in MAP in CRF rats, suggesting that oxidative stress is increased in the region probably independent of AT_1_ Ang II receptors activation.

CRF is associated with depressed SOD and elevated NADPH oxidase expression, which can contribute to oxidative stress by increasing superoxide anion. A depression in SOD expression was reported previously [6] for the same CRF model that we used, that could be correlated with the observed diminution in the activity of SOD and catalase (CAT), which can contribute to oxidative stress by increasing superoxide anion and hydrogen peroxide generation.

Superoxide derived from NAD(P)H oxidase avidly reacts with and inactivates NO and, thereby, modulates its bioavailability [6]. The availability of biologically active NO in a given tissue depends not only on the rate of its production, but also on the rate of its inactivation by superoxide (NO + O_2_^−^ONOO^−^) [7].

The mechanisms by which the increase in ROS in the RVLM increased blood pressure are not known. These responses might be mediated by an interaction between superoxide and NO. Superoxide anions react rapidly with NO, forming peroxynitrite and decreasing the bioavailability of NO in the RVLM. This mechanism contributes to the increase in sympathetic nerve activity and hypertension [8].

The involvement of Ang II is believed to decline, whereas oxidative stress increases during the progression of renovascular hypertension. The administration of an AT_1_ receptor blocker indeed reduced the MAP both during the early and the chronic phase in renovascular hypertension but failed to improve either the renal hemodynamics or oxygenation during the early phase. The acute administration of tempol is more effective in lowering MAP in chronic renovascular hypertension than blockade of the renin-angiotensin system with ARB or angiotensin-converting enzyme (ACE) inhibitor [9]. In the present study, the increased oxidative stress within the RVLM is probably independent of AT_1_ Ang II receptors based on functional and molecular results.

A previous study described that subtotal nephrectomy induced a significant increase in TBARS of 144% and SOD activity was significantly decreased to 55% in the kidney [10]. 

As the activation of the RVLM neurons increases blood pressure, mediated by increases in vascular peripheral resistance, as well as by cardiac contractility and secretion of catecholamines [11, 12] changes in the neuronal excitability in this region may be a mechanism involved in the pathophysiology of arterial hypertension. In fact, electrophysiological studies in spontaneously hypertensive rats **(**SHRs) have confirmed increases in the firing rates of RVLM barosensitive neurons containing excitatory amino acids and terminating in the intermediolateral cell column of the spinal cord, where the preganglionic sympathetic neurons are located [13, 14].

 In the present study we found an increase in the NADPH subunits in the RVLM and a decrease in MAP in response to tempol. However, glutamate into the RVLM increased MAP more intensively in control than in CRF rats. Changes in the neuronal excitability in response to local alterations in neurotransmitters may be involved in such response. Indeed in CRF rats we found a decrease in nNOS expression (data not shown) within the RVLM that might be responsible for the reduced RVLM response to glutamate.

Oral administration of GABA in SHR leads to a decrease in blood pressure accompanied by a decrease in plasma renin activity. The mediation of this hypotensive effect appears to involve the renal nerves by a decrease in renal sympathetic nerve activity [15]. GABA administration ameliorated renal function (serum urea nitrogen, Cr, and urinary protein) in nephrectomized rats (5/6) [16]. In the present study, GABA into the RVLM induced larger decrease in MAP in CRF rats compared to controls, suggesting that the sympathetic vasomotor tone is a major mechanism leading to hypertension. Oxidative stress within the RVLM is one of the sources of increased RVLM activity.

## 4. Conclusion

Altogether, the results suggest that the hypertension in CRF rats is in part mediated by increased oxidative stress in the RVLM.

## Figures and Tables

**Table 1:** PCR primer sequences for Real Time quantitative PCR.

Gene	Primer	Sequence (5′ to 3′)	Product size (bp)	Annealing Temperature
AT_1_	5′	ATG-CCA-GTG-TGT-TTC-TGC-TC	244	58,0°C
		CCA-ATG-GGG-AGT-GTT-GAG-TT		
*β*-actin	5′	CCT-CTA-TGC-CAA-CAC-AGT-GC	191	56,9°C
		ACA-TCT-GCT-GGA-AGG-TGG-AC		
gp91^phox^	5′	AGT-GGT-TCG-CAG-ACC-TGC-TG	239	57°C
		TCT-GGT-GTT-GGG-GTG-TTG-AC		
p47^phox^	5′	GTC-TGA-GGG-TGA-AGC-CAT-CG	211	59°C
		CCG-AGA-ACG-CTG-GTG-GAT-GC		

Angiotensin II type 1 (AT_1_), *β*-actin (housekeeping), NAD(P)H subunits (p47^phox^ and gp91^phox^).

**Table 2:** Values of mean arterial pressure increase (delta of MAP) in response to Candesartan microinjection into the RVLM in hypertensive (CRF) and control (C) rats.

	MAP (mmHg)	
	CRF	C
Candesartan 0.25 nmol (*n* = 5)	59 ± 7	50 ± 4
Candesartan 0.5 nmol (*n* = 5)	57 ± 9	62 ± 3
Candesartan 1.0 nmol (*n* = 5)	26 ± 5	64 ± 8
